# Progress in pathogenesis research of *Ustilago maydis*, and the metabolites involved along with their biosynthesis

**DOI:** 10.1111/mpp.13307

**Published:** 2023-02-17

**Authors:** Chunyan Yu, Jianzhao Qi, Haiyan Han, Pengchao Wang, Chengwei Liu

**Affiliations:** ^1^ Key Laboratory for Enzyme and Enzyme‐Like Material Engineering of Heilongjiang, College of Life Science Northeast Forestry University Harbin China; ^2^ Shaanxi Key Laboratory of Natural Products & Chemical Biology, College of Chemistry & Pharmacy Northwest A&F University Yangling China

**Keywords:** biosynthesis, effectors, pathogenesis, surfactant, *Ustilago maydis*

## Abstract

*Ustilago maydis* is a pathogenic fungus that causes corn smut. Because of its easy cultivation and genetic transformation, *U. maydis* has become an important model organism for plant‐pathogenic basidiomycetes. *U. maydis* is able to infect maize by producing effectors and secreted proteins as well as surfactant‐like metabolites. In addition, the production of melanin and iron carriers is also associated with its pathogenicity. Here, advances in our understanding of the pathogenicity of *U. maydis*, the metabolites involved in the pathogenic process, and the biosynthesis of these metabolites, are reviewed and discussed. This summary will provide new insights into the pathogenicity of *U. maydis* and the functions of associated metabolites, as well as new clues for deciphering the biosynthesis of metabolites.

## Graphical abstract

*Ustilago maydis* is a common biotrophic plant‐pathogenic fungus that causes serious economic losses. We summarize the research on maize pathogenesis, and the metabolites related to *U. maydis* infection and their biosynthesis.

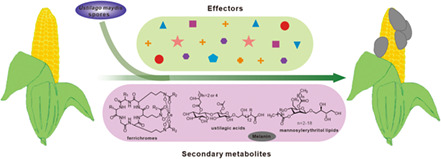

## 1 INTRODUCTION

*Ustilago maydis*, a basidiomycete fungus, is a common biotrophic phytopathogenic fungus capable of infecting all aerial organs of corn plants. It is parasitic exclusively on maize (*Zea mays*), one of the major cereal crops in the world, and its ancestor, teosinte (*Z. mays* subsp. *mexicana*). This disease can hinder the growth of plants and reduce the yield, which leads to serious economic losses. The *U. maydis–*maize interaction process has effects at the cellular and molecular levels, leading to host cell physiology and metabolic disorders, manifesting as characteristic tumours formed by different host organs (Luo et al., 2020). The life cycle of *U. maydis* involves the germination of diploid teliospores on the host, followed by mating between haploid meiotic progeny to form invasive dikaryotic mycelia. The hyphae then form appressoria to invade host tissues, followed by extensive proliferation, tumour induction, and eventual formation of pigmented teliospores (Kämper et al., 2006). To successfully colonize its host, *U. maydis* has developed various strategies to respond to the host, such as releasing effectors in response to the reprogramming of host metabolism (Cui et al., 2015; Djamei & Kahmann, 2012). To date, hundreds of effectors have been discovered, and the functions of some of the core effectors have been thoroughly characterized. The effectors act in multiple ways on different targets, such as suppressing plant immunity, manipulating plant physiology, and being recognized by host defence mechanisms, thus promoting pathogen infestation, expansion, and colonization. In the narrow sense, effectors are proteins secreted by pathogens into the extracellular and intracellular spaces of host plants (Duplessis et al., 2011; Gan et al., 2013; Giraldo et al., 2013; Saitoh et al., 2012). In the broad sense, effectors are proteins and small molecules that alter the structure and function of host cells, thereby promoting the colonization of pathogens (Horbach et al., 2011). The rapid development of omics technologies has facilitated the discovery and functional characterization of an increasing number of effectors. The increasing deciphering of the *U. maydis* genome provides more valuable information for revealing the pathogenic mechanism (Han & Kahmann, 2019; Lanver et al., 2017; Redkar et al., 2017). Due to its highly divergent development and reverse genetic adaptations, *U. maydis* provides a model system for the genetics and cell biology of smut fungus, as well as for studies on the interaction between living trophic plant pathogens and plants (Zuo et al., 2019).

**FIGURE 1:**
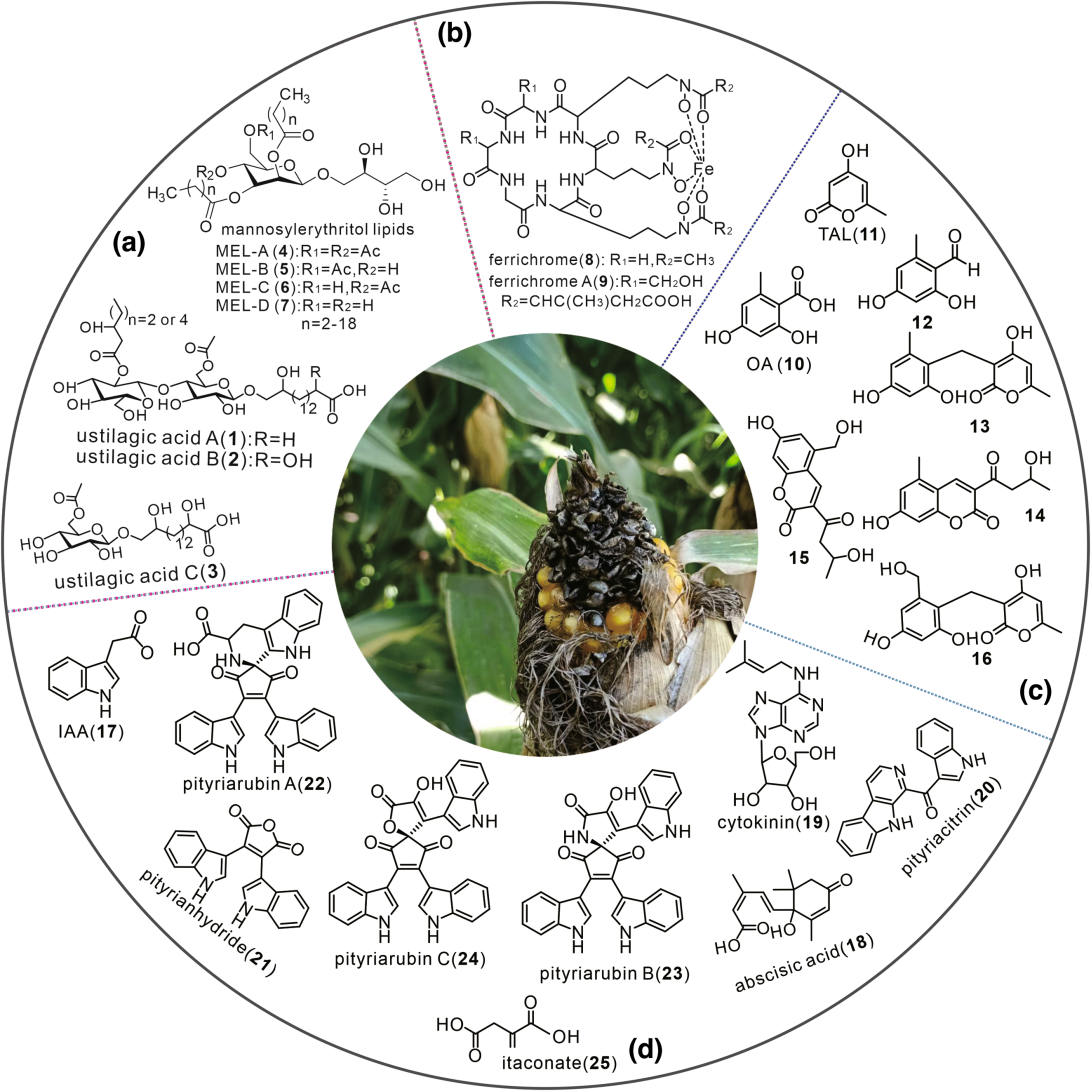
Structural diversity of secondary metabolites of *Ustilago maydis*. (a) Ustilagic acids and mannosyl erythritol lipids (MELs), (b) ferrochromes, (c) polyketides, (d) miscellaneous compounds. IAA, indole‐3‐acetic acid; OA, orsellinic acid; TAL, triacetic acid lactone.

Although *U. maydis* is well known as a maize pathogen, its metabolites have many biological activities. Chemical composition investigation and activity identification studies have shown that *U. maydi*s‐derived ustilagic acids (UAs, 1–3 in Figure 1) (Kahmann & Kamper, 2004; Yang et al., 2013) and mannosyl erythritol lipids (MELs, 4–7 in Figure 1) contain uncommon glycolipids of cellobiose and erythrose with biosurfactant and antifungal activity (Teichmann, Lefebvre, et al., 2011). These compounds not only assist in the process of *U. maydis* appressoria adhesion to the plant surface, but also prevent damage to plants by other fungi (Kuiper et al., 2004). In addition, *U. maydis* was also found to yield other metabolites, such as ferrichromes (8–9 in Figure 1) (Neilands, 1981; Winterberg et al., 2010) and melanin (Reyes‐Fernandez et al., 2021). Herein, we summarize advances in the pathogenesis of *U. maydis*, as well as the metabolites and their biosynthesis associated with corn smut infection. This review provides a specific perspective on the pathogenicity of *U. maydis* that will contribute to a comprehensive understanding of corn smut.

## 2 THE LIFE CYCLE OF *U. maydis* AND ITS PATHOGENICITY

**FIGURE 2:**
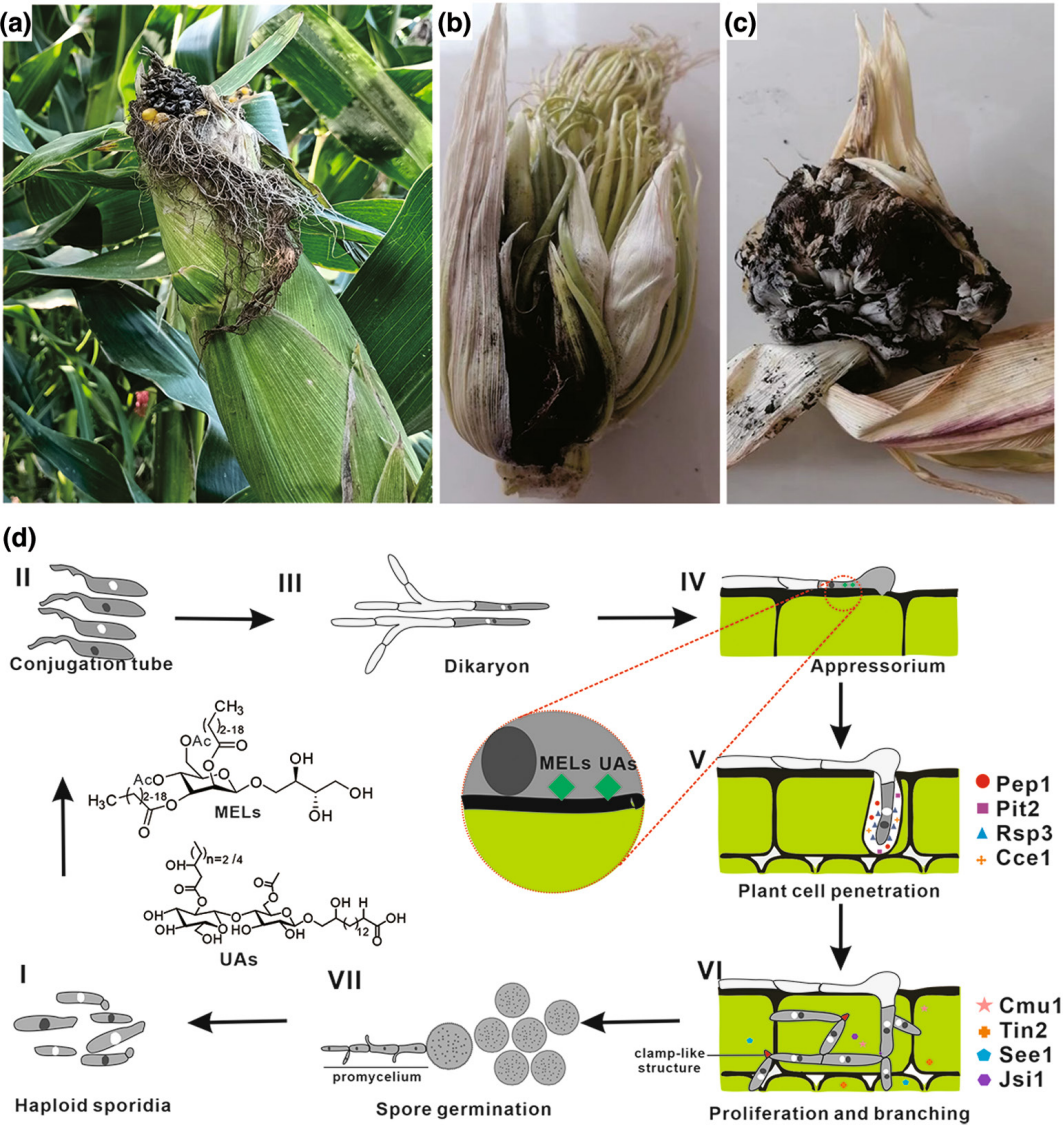
Corn smut and infection of *Ustilago maydis*. (a) Ripe corn cobs mildly infected with corn smut, (b) severely infected ears of corn smut, (c) tissue filled with mature corn smut spores, and (d) the life cycle of *U. maydis*. Effectors: Pep1 (protein essential during penetration 1), Pit2 (protein important for tumours 2), Rsp3 (repetitive and secreted protein 3), Cce1 (cysteine‐rich core effector 1), Cmu1 (chorismate mutase 1), Tin2 (tumour‐inducing effector 2), See1 (seedling efficient effector 1), and Jsi1 (jasmonate/ethylene signalling inducer 1). MEL, mannosyl erythritol lipid; UA, ustilagic acid.

Many fungal plant pathogens cause drastic morphological changes in host organs due to host cell proliferation and overgrowth (Wildermuth, 2010). The smut fungi Ustilaginales have been found to cause extreme changes in host tissue morphology (Luttrell, 1981). *U. maydis* has become a model microorganism used to study the mechanisms of interactions between biotrophic fungi and plants (Bölker, 2001). Considered as one of the top 10 plant fungal pathogens, *U. maydis* can infect all aboveground organs of the maize plant, including seedlings, ears, and adult leaves, and induce tumour (commonly known as maize black truffles) formation and become a significant threat to modern maize productivity (Dean et al., 2012). The parasitism of *U. maydis* does not lead to the death of maize plants (Figure 2a). However, when the infection is severe, maize does not spit ears (Figure 2b), and further forms massive tumour tissue (Figure 2c). To successfully colonize hosts, *U. maydis* has developed several strategies including evasion of host recognition, interference with plant defence responses, and reprogramming of host metabolism (Redkar et al., 2017).

The two‐stage life cycle (Figure 2d) of *U. maydis* is closely related to its infection process, which has been revealed by numerous investigations. First, haploid spores undergo saprophytic growth and germinate on specific substrates to produce yeast‐like colonies. However, this form is not pathogenic (Figure 2d, I). Then, the compatible haploid cells form a conjugation tube (Figure 2d, II) and fuse to form an invasive binucleate mycelium (Figure 2d, III) (Kahmann & Schirawski, 2007). In the early stage of infection, the ends of the binucleate hyphae of *U. maydis*, known as appressoria, swell and begin to penetrate the epidermal cells of growing maize (Figure 2d, IV) (Lanver et al., 2014; Snetselaar & Mims, 1993). These filaments differentiate into infection structures known as appressoria. After penetration of the epidermal layer of the plant, the cell cycle arrest ceases and clamp‐like structures ensure the correct separation of two different nuclei and maintain the dikaryotic state in the growing hyphae (Lanver et al., 2017). The extracellular mycelium grows between cells without causing visible disease, while the intracellular mycelium is tightly surrounded by the plant plasma membrane, forming a living nutrient interface that facilitates the exchange of nutrients and signalling molecules, including various proteins (Matei & Doehlemann, 2016) (Figure 2d, V). This process involves the secretion of hundreds of effectors by *U. maydis* in various ways to suppress the plant's innate immune system and manipulate host metabolism (Cui et al., 2015; Djamei & Kahmann, 2012; Han & Kahmann, 2019). Subsequently, the mycelium proliferates massively in the plant foliar tissue, vascular system, and surrounding cavities, resulting in the formation of plant tumours (Figure 2d, VI). Next, extracellular hyphae form large aggregates in the cavities between plant tumour cells, the nuclei of binucleate mycelium cells fuse, and the mass proliferating hyphae break off and form pigmented aggregates of spores (Matei & Doehlemann, 2016). When the tumours dry up and rupture, the spores are released (Figure 2d, VII) and germinate under suitable conditions. The nuclei of diploids undergo meiosis and germination to form promycelium and haploid spores. The entire life cycle of *U. maydis* is strictly dependent on the plant, and usually takes about 2 weeks (Lanver et al., 2018).

## 3 EFFECTORS INVOLVED IN PATHOGENIC PROCESSES

Maize undergoes a series of physiological and biochemical changes in response to *U. maydis* infection (Wang et al., 2007). Doehlemann et al. investigated maize's defensive response to *U. maydis* infection using transcriptome and metabolome analysis, revealing that secondary metabolite production was activated, cell division mechanisms were increased, and pathogenesis‐related genes were induced (Doehlemann, Wahl, Horst, et al., 2008). Zou et al. analysed transcriptomic data on the early response to *U. maydis* infection of maize, revealing specific changes in maize cells during tumour formation (Zou et al., 2022). In brief, when infected with *U. maydis*, maize strengthens its defensive mechanism through physiological changes (Djamei et al., 2011; Li et al., 2016). However, during an infection, *U. maydis* suppresses the plant's defensive response by secreting effectors (Win et al., 2012). Effectors released by pathogens are secreted proteins that affect the structure and function of host cells, thus facilitating the success of the pathogen infestation process.

### 3.1 The type of function of the effectors

Most effectors are secreted proteins generated by pathogenic strains, and genome sequencing has tremendously aided in effector discovery and identification. The effectors have different functions due to different locations, and apoplastic effectors play a role in the interaction area between fungal hyphae and the host (Stotz et al., 2014). Generally, the targets of these apoplastic effectors exist in the first layer of the plant defence response. Most apoplastic effectors are plant cell wall‐degrading enzymes (PCWDEs), which can destroy the structure of host cell walls and facilitate hyphae invasion (Chen et al., 2021; Quoc & Chau, 2017). The effectors located in the cytoplasm can regulate host resistance by regulating the maturation and localization of plant resistance proteins (Qi et al., 2019). In addition, interfering nucleoprotein can directly regulate the transcription of many immune‐related genes. Some nuclear effectors have been well characterized in plant‐pathogenic fungi (Wu et al., 2022).

The decryption of *U. maydis* 521 genomic information revealed that 18.6% of the genes encoding secreted proteins are distributed in 12 clusters of the genome, ranging from three to 26 genes per cluster. These genes were up‐regulated in expression on *U. maydis* infestation of plants (Kämper et al., 2006). *U. maydis* can produce 467 putative secreted effector proteins, which can promote the colonization of *U. maydis* to the host (Lanver et al., 2018). The finding of these secreted proteins has established the groundwork for further research into *U. maydis* effectors. Furthermore, effectors respond to particular processes happening in the host, such as host reprogramming, to increase infection (Doehlemann et al., 2014; Lanver et al., 2017). Because *U. maydis* lacks specialized feeding structures, signal exchange and nutrient absorption must occur via biotrophic interfaces (Brefort et al., 2009). Despite substantial transcriptome research revealing the spatiotemporal‐specific expression of *U. maydis* effector genes (Lanver et al., 2018; Mine et al., 2018), only a few critical effectors have been functionally characterized (Table 1). These effectors play an important role in inhibiting the accumulation of reactive oxygen species (ROS), promoting the virulence of *U. maydis*, regulating phytohormone metabolism, and inducing plant tissue swelling.

**TABLE 1:** Key effectors of *Ustilago maydis* and their function.

Effector	Function	Known target	Localization	Parasitic stage	Reference
Pep1	Suppression of the plant's oxidative	POX12	Live nutrition interface/apoplastic	Weakening the defence reactions of host plant	Doehlemann et al. (2009); Hemetsberger et al. (2012); Hemetsberger et al. (2015)
Rip1	Suppression of the reactive oxygen species burst	Zmlox3	Nucleus		Saado et al. (2022)
Rsp3	Promotes virulence	AFP1, AFP2	Live nutrition interface/apoplastic	Weakening the defence reactions of host plant	Ma et al. (2018)
Jsi1		TPL/TPRs	Nucleus		Darino et al. (2021)
Cce1		–	Apoplastic		Seitner et al. (2018)
Nkd1		TPL/TPRs	Nucleus		Navarrete et al. (2022)
Vp1	Promotes fungal colonization and virulence of *U. maydis*	–	Apoplastic/cytosol of plant cells/nucleus		Hoang et al. (2021)
See1	Reactivation of plant DNA synthesis	SGT1	Plant cytoplasm/nucleus	Induction of tumour	Redkar, Hoser, et al. (2015); Redkar, Villajuana‐ Bonequi, et al. (2015)
Pit2	Inhibits maize apoplastic cysteine proteases	CP2, CP1A/B, XCP2	Live nutrition interface/apoplastic	Weakening the defence reactions of host plant	Doehlemann et al. (2011); Mueller et al. (2013)
Cmu1	Suppresses salicylic acid synthesis	ZmCm1, ZmCm2	Live nutrition interface/plant cytoplasm		Djamei et al. (2011); Han et al. (2019)
Tin2	Promotes anthocyanin biosynthesis	ZmTTK1	Cytosol of plant cells	Hyphal proliferation	Tanaka et al. (2014)
Cpl1	–	–	Cell wall of *U. maydis*	Weakening the defence reactions of host plant	Weiland et al. (2022)
Lep1	Induction of the morphological changes associated with spore formation	–	Discrete areas below the tips in biotrophic hyphae of *U. maydis*	Induction of tumour	Fukada et al. (2021)

*Note*: –, not available.

Studies have shown that *U. maydis* can inhibit the production of ROS by secreting the following effectors. Pep1 plays a key role in the infection of maize and suppresses the plant's oxidative burst by attaching to the plant's ectoplasmic peroxidase POX12. When *U. maydis* infects maize plants, the Pep1‐encoding gene inactivation induces an oxidative burst in the host plant, resulting in H_2_O_2_ build up in the host cell wall and hence a severe host immunological response (Doehlemann et al., 2009; Hemetsberger et al., 2012). Hemetsberger et al. (2015) categorized Pep1 as a phylogenetically conserved core effector of *U. maydis*, noting that this effector may play an essential role in biotrophic smut fungus pathogenicity. Rip1 is an effector whose function has not been fully elucidated. It inhibits the function of pathogen‐associated molecular pattern (PAMP)‐triggered ROS, thereby reducing host immunity (Saado et al., 2022). Late effector protein 1 (Lep1) is a novel core effector that is highly expressed during tumour formation (Fukada et al., 2021).

The core effector Rsp3 is a critical virulence factor that protects mycelium from maize DUF26‐domain family proteins (AFP) proteins (Ma et al., 2018). Another core effector, Jsi1, interacts with the plant corepressor family Topless/Topless related (TPL/TPRs) proteins by increasing the transcription of the ethylene response factor (ERF) branch of the jasmonate/ethylene (JA/ET) signalling pathway, thereby promoting virulence and suppressing the host immune system (Darino et al., 2021). Cce1 is a recently identified core effector that is localized in the plasmodial ectodomain; however, its target has yet to be identified (Seitner et al., 2018). Naked1 (Nkd1) is an effector localized in the nucleus that was identified by Navarrete et al. (2022). By binding to transcriptional co‐repressors TPL/TPRs, Nkd1 not only blocks auxin signalling and recruitment of transcriptional repressors but also leads to activation of auxin and JA signalling and promotes susceptibility to (semi‐)biotrophic pathogens (Navarrete et al., 2022). Hoang et al. (2021) found that virulence promoting 1 (Vp1) is another novel secreted effector protein. Vp1 is localized to the plasmodial ectodomain and promotes its own virulence and ability to colonize the host (Hoang et al., 2021).

*U. maydis* can also secrete other effectors to ensure its successful colonization on the host. The translocation effector See1 is transferred to the maize cytoplasm and nucleus by the mycelium. In the cytoplasm, it interacts with SGT1 in maize and interferes with mitogen‐activated protein kinase (MAPK)‐induced phosphorylation of SGT1. In the nucleus, See1 is required for the reactivation of DNA synthesis. Finally, it promotes the formation of tumours in seedling leaves (Redkar et al., 2015). The effector Pit2 inhibits the activity of the key virulence target maize cysteine protease (CP1A, CP1B, CP2, and XCP2), which is required for the formation of tumours, while mutant *pit2* strains induce a strong defence response in the host, suppressing mycelial spread and tumour formation (Doehlemann et al., 2011; Mueller et al., 2013). Cmu1 is a secreted effector (Djamei et al., 2011) that can be translocated into plant cells to interact with two maize chorismate mutases, ZmCm1 and ZmCm2, and inhibit the synthesis of salicylic acid (SA) by regulating chorismite homeostasis. The Δ*cmu1* mutant strain inhibited tumour formation and pathogenicity (Djamei et al., 2011; Han et al., 2019). The effector Tin2 interacts intracellularly with the maize protein kinase ZmTTK1 to promote anthocyanin bioproduction and reduce lignin biosynthesis, thereby preventing lignification at the infection site. When Tin2 is knocked out, ZmTTK1 undergoes ubiquitination, which promotes lignin synthesis and thus weakens mycelial proliferation (Tanaka et al., 2014). Cerato‐platanin‐like (Cpl1) is an active effector in the early stages of plant infection and localizes to the cell wall of *U. maydis*, specifically enriching cell‐wall degrading and decorating proteins during infection (Weiland et al., 2022).

Effectors are involved in almost all processes of corn smut infection of plants. They are constantly under selection pressure from the host immune system. As a result, genes encoding effectors are the fastest evolving genes in pathogen genomes (Stergiopoulos et al., 2007). Because of the lack of identifiable structural domains and functional redundancy among effectors, functional identification of effectors is difficult. As a consequence, the functions of a large number of effectors (Table S1) are either unknown or only partially known. These effectors function in the interaction between *U. maydis* and the host, thereby promoting *U. maydis* infection and colonization of maize plants.

### 3.2 Synthetic regulation of effectors

When the yeast‐like haploid spores are transformed into dikaryotic hyphae, *U. maydis* is able to parasitize living plants. This dimorphic transformation is closely related to the process of infecting host plants, so effector expression regulation is essential for hyphal development (Lanver et al., 2017). The A and B alleles of the quadruple mating type system control this transformation process, with the A site controlling haploid spore mating and the B site determining the pathogenicity of the dikaryotic hyphae. Following the formation of the dikaryotic hyphae, the appressoria form in the proper position and begin the colonization process. Following that, the bE/bW heterodimer regulates the hyphal formation and pathogenic development (Brachmann et al., 2001; Müller et al., 2003). Pheromone responses, bE/bW‐mediated transcriptional regulatory networks, and surface signal sensing pathways all influence *U. maydis* effector expression (Müller et al., 2003). There have been numerous research reports on the regulation and regulatory network of effector synthesis (Brefort et al., 2009; Lanver et al., 2017). The key regulatory genes that are helpful to the *U. maydis* infection process are shown in Table 2.

**TABLE 2:** Key regulatory genes in the pathogenic process of *Ustilago maydis.*

Stage	Key regulatory genes	Function	Reference
**Stage I**: Haploid sporidia	*hgl 1*	Inhibit the germination of yeast‐like basidiospores	Dürrenberger et al. (2001)
	*ust 1*	Promote the development of yeast‐like basidiospores to primary hyphae	García‐Pedrajas et al. (2010)
**Stage II:** Conjugation tube formation	*kpp 4* /*ubc4*, *fuz7*/*ubc5*, *kpp2*/ubc*3*	Elicits formation of conjugation hyphae	Müller et al. (2003)
**Stage III**: Dikaryotic hyphae formation	*a/b* locus, *pra1*/*2*, *rbf1*	The pheromone receptor Pra on the surface of the host cell senses the pheromone and transmits the signal to cAMP and MAPK signalling pathways, and forms dikaryotic hyphae	Heimel, Scherer, Vranes, et al. (2010); Kaffarnik et al. (2003)
**Stage IV**: Appressorium formation	*sho 1*, *msb2*, *pmt4*, *hdp2*, *biz1*	The appressorium adheres to the surface of the host plant and regulates the formation of the appressorium	Fernández‐Alvarez et al. (2009); Lanver et al. (2010)
**Stage V**: Penetratio plant epidermis and weakening host defence	*kpp 6*, cluster 5B, *pep1*, *yap1*	Regulatory factors respond to the host's defence response	Brachmann et al. (2003); Hemetsberger et al. (2012); Kämper et al. (2006); Molina and Kahmann (2007)
**Stage VI**: Multiplication and branching in the host	*clp 1*, cluster 19A, *str1*, *hxt1*, *gas1*	The mycelium proliferates rapidly in the host, causing the host tissue to form a tumour	Brefort et al. (2014); Heimel, Scherer, Schuler, et al. (2010); Schirawski et al. (2005); Schuler et al. (2015); Wahl et al. (2010)
**Stage VII**: Tumour formation and production of teliospores	*gpa 3*, *ubc1*, *rtf1*, *ust1*, *ssp1*, cluster 19A, *hgl1*, *umv1*, *umv2*	The formation of mature spores in the host tumour tissue indicates that the next life cycle from spores has begun	Banuett (1991); Dürrenberger et al. (2001); García‐Pedrajas et al. (2010); Gold et al. (1997); Huber et al. (2002); Krüger et al. (2000); Lanver et al. (2014); Schmitz et al. (2018)

Pheromone receptors Pra1 and Pra2 on the surface of host cells can sense pheromones and transmit signals to the cAMP and MAPK signalling pathways (Müller et al., 2003). These two signalling pathways further transmit the signal to the transcription factor Prf1, which causes the b site to be expressed, resulting in the formation of a bE/bW heterodimer (Kaffarnik et al., 2003). The bE/bW heterodimer can regulate the downstream transcription factor network through the transcription factor Rbf1 (Heimel, Scherer, Vranes, et al., 2010).

Early in the infection process, gene regulation of the zinc finger transcription factor Rbf1 is known to be required for the initial steps of pathogenic development, including hyphal formation, induction of G2 cell cycle arrest, and stress‐mediated plant infiltration (Heimel, Scherer, Vranes, et al., 2010). The transcription factors Hdp2 and Biz1 downstream of Rbf1 are involved in the multiple regulation of effectors during the biotrophic establishment stage, and 228 effector genes, including important effectors such as Pep1, Pit2, Rsp3, and See1, are up‐regulated (Brefort et al., 2014; Lanver et al., 2017; Schmitz et al., 2018).

Sho1 and Msb2 sense signals such as hydrophobic structures and cutin monomers on the plant surface, and transmit the signals to Hdp2 and Biz1, thereby promoting hyphae formation of appressoria and pathogenicity (Lanver et al., 2010). The gene *pmt4* encoding *O*‐mannosyltransferase plays a key role in the formation of appressoria. Deleting the *pmt4* gene resulted in a significant reduction in the formation of appressoria at the tip of the dikaryon. Pmt4 may activate the activity of Msb2 by *O*‐mannosylating the protein and promote the formation of appressoria (Fernández‐Alvarez et al., 2009).

Fox1 is required to suppress plant defence responses. It can regulate the expression of 141 genes, including 38 possible effector‐encoding genes (García‐Pedrajas et al., 2010; Xia et al., 2021). There are also regulatory genes or gene clusters that play an important role in mycelial penetration of the plant epidermis and weakening of host defences, such as *kpp6* (Brachmann et al., 2003), cluster 5B (Kämper et al., 2006), and *pep1* (Hemetsberger et al., 2012; Molina & Kahmann, 2007).

In the late stage of infection, the main functions of effectors are to regulate processes such as tumour maturation and black teliospore production. There are few studies on the transcription factors regulating these late events. Ros1 down‐regulated several critical effectors involved in the early colonization process and up‐regulated the late expression of 51 novel effectors (Tollot et al., 2016). The transcription factor Nlt1 is also an important regulator, but only in lobe tumours (Lanver et al., 2018). At this stage, some regulatory genes or gene clusters can regulate the infection of *U. maydis*, such as *clp1* (Heimel, Scherer, Schuler, et al., 2010), cluster 19A (Brefort et al., 2014), and *str1* (Wahl et al., 2010) that can make hyphae proliferate and branch in the host; and *gpa3* (Krüger et al., 2000), *ubc1* (Gold et al., 1997), *rtf1* (Banuett, 1991), *ust1* (García‐Pedrajas et al., 2010) and *ssp1* (Huber et al., 2002) that make the host form tumour‐like tissue and produce spores.

The secretion of effectors during the infection process brings extensive pressure to the endoplasmic reticulum. After the effectors are regulated by transcription factors, they undergo protein modification in the endoplasmic reticulum and the Golgi apparatus. The unfolded protein response (UPR) is a conserved eukaryotic signalling pathway that detects misfolded proteins in the endoplasmic reticulum. There is significant crosstalk between the UPR pathway and the bE/bW regulatory cascade (Lanver et al., 2018; Tollot et al., 2016). After *U. maydis* hyphae penetrate the plant epidermis, the UPR is activated immediately and participates in the regulation of the next step of gene regulation (Heimel, Scherer, Schuler, et al., 2010). After modification, effectors are secreted into the apoplast and host cells to function. For example, *U. maydis* secretes the effector Cce1 to attenuate callose deposition in the cell wall (Matei & Doehlemann, 2016), and the metalloprotease Fly1 can destroy chitinases in host plants (Ökmen et al., 2018). The conserved effector protein Erc1 binds to host cell wall components and exhibits 1,3‐β‐glucanase activity, which attenuates the β‐glucan necessary for the induced defence response (Ökmen et al., 2022).

### 3.3 Effector and omics research

*U. maydis* has become one of the most dangerous diseases in maize production due to the rampant occurrence of smut diseases and the decreasing resistance of crop varieties. Genome sequencing of *U. maydis* is indispensable for a comprehensive understanding of the genetic information and pathogenesis. Kämper et al. (2006) published the first genome sequence of *U. maydis.* The genome size of 20.5 Mb is smaller than that of most plant pathogens. So far, 38 *U. maydis* genomes have been sequenced and published (Table S2), which not only provides valuable clues for in‐depth interpretation of the genome of *U. maydis*, but also provides an in‐depth understanding of the secondary metabolites.

Recent developments in transcriptomics, as well as comparative genomics, have facilitated the in‐depth study of effectors, and many studies are no longer limited to the functional analysis of individual effectors, but rather to the comparative and in‐depth analysis of the functions of proximate effectors and their evolutionary history (Dutheil et al., 2016).

The discovery of the effectors' functions contributes significantly to the understanding of the corn smut infection mechanism. Secondary metabolites produced by *U. maydis*, in addition to effectors, increase its pathogenicity. MELs and UAs are presumed to help appressoria adhere to the smooth hydrophobic surfaces of host plants (Haskins, 1950; Kuiper et al., 2004; Lemieux & Charanduk, 1951). Thus, secretion of metabolites like MELs and UAs are thought to play important supporting roles in the processes of hyphal attachment and infestation of plant surfaces (Feldbrügge et al., 2013). Additionally, siderophore (Mei et al., 1993) and melanin (Islamovic et al., 2015) were found to be involved in the infection processes of *U. maydis*.

## 4 SECONDARY METABOLITES INVOLVED IN PATHOGENIC PROCESSES AND THEIR BIOSYNTHESIS

The production of secondary metabolites by fungi is not without merit. For example, the polyene compounds secreted by mushrooms can inhibit insect bites and are a class of efficient chemical defence molecules (Brandt et al., 2017). *U. maydis* also produces some specific secondary metabolites to facilitate its infection of maize. To date, 25 secondary metabolites have been reported, including UAs (1–3), MELs (4–7), ferrichromes (8–9), melanin precursor (11–16), and other compounds (17–25) (Figure 1), some of which are associated with the pathogenesis of corn smut.

### 4.1 UAs and their biosynthesis

UAs are composed of cellobiose linked to 15,16‐dihydroxypalmitic acid (ustilagic acid A; 1 in Figure 1) or 2,15,16‐trihydroxypalmitic acid (ustilagic acid B; 2) via *O*‐glycosidic bonds, while cellobiose is composed of acetyl modified and short‐chain fatty acids (Haskins, 1950; Lemieux, 1951; Lemieux et al., 1951; Lemieux & Charanduk, 1951). Ustilagic acid C (3) was isolated as a new component from *U. maydis* treated with chemical epigenetic modifiers (Yang et al., 2013). UAs were the first glycolipids to be discovered with cellobiose, a rare moiety in natural products. UAs are therefore also regarded as characteristic metabolites of *U. maydis*.

**FIGURE 3:**
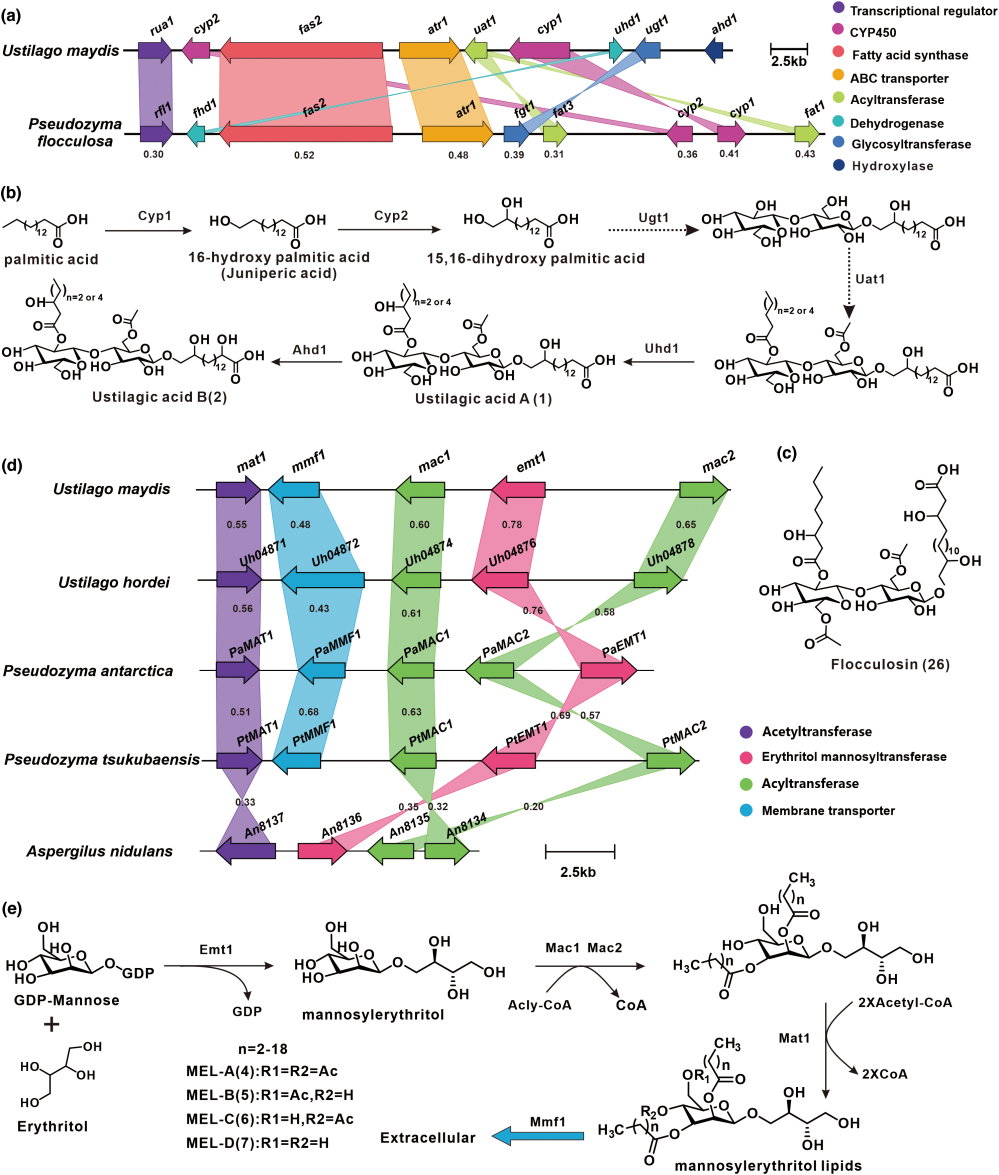
The biosynthesis of ustilagic acids (UAs) and mannosyl erythritol lipids (MELs). (a) Biosynthetic gene cluster (BGC) for UAs, (b) the proposed biosynthetic pathway of UA, (c) the chemical structure of flocculosin (26), (d) BGCs of MELs from *Ustilago maydis* and other species, and (e) the proposed pathway of MELs.

Teichmann et al. discovered that under nitrogen starvation conditions, all candidate genes related to UA biosynthesis were significantly up‐regulated, thus deducing the biosynthetic gene cluster (BGC) responsible for UAs (Figure 3a) (Teichmann et al., 2007, 2010; Teichmann, Lefebvre, et al., 2011). The BGC contained nine genes, including one transcriptional regulator *rua1* and eight functional genes (Figure 3a). The expression of all of the genes is regulated by Rua1. The *cyp1* mutant restored UA production after supplementation with 16‐hydroxypalmitate, thus indicating that Cyp1 is responsible for the terminal hydroxylation of palmitate. A *cyp2* mutant produced UA lacking hydroxyl groups, indicating that Cyp2 is responsible for the minor terminal hydroxylation of palmitate. The *ahd1* mutant secretes UA lacking α‐hydroxyl groups, indicating that Ahd1 is responsible for the α‐hydroxylation of UA (Teichmann et al., 2007). Knockout of *cyp2* revealed that the UA produced by the *cyp2* mutant strain lacked the hydroxyl group, suggesting that Cyp2 is responsible for the secondary terminal hydroxylation of palmitic acid. When *rua1* is knocked out, UA is not produced. The biosynthetic pathway of UAs is summarized in Figure 3b.

Flocculosin (26) was discovered as a biocontrol agent, structurally similar to UAs, in *Pseudozyma flocculosa* (Teichmann, Labbé, et al., 2011) (Figure 3c). The difference is that flocculosin contains an additional acetyl group for cellobiose (Teichmann, Labbé, et al., 2011). The flocculosin BGC contains 10genes, eight highly homologous to their UA BGC counterparts (Teichmann, Labbé, et al., 2011).

### 4.2 MELs and their biosynthesis

MELs are well‐known biosurfactants and were discovered in the metabolites of *Ustilago* sp. as early as 1955 (Haskins et al., 1955). Structurally, the backbone of MELs is a disaccharide composed of mannosyl and erythritol, and C4 and C6 of the mannosyl moiety are linked to short‐chain and medium‐chain fatty acids through ester groups (Deinzer et al., 2019). MELs are frequently classified as A, B, C, and D (4–7 in Figure 1) due to differences in the number of acetyl groups attached to C4 and C6, as well as their appearance order on thin‐layer chromatography. MEL‐A (4) represents a diacetylated compound (over 70% of MELs), while MEL‐B and MEL‐C are monoacetylated at C6 and C4, respectively, and the fully deacetylated structure is called MEL‐D (Rau et al., 2005).

Hewald et al. (2006) used the homologous recombination method (Kamper, 2004) for *U. maydis* genetic manipulation previously developed to knock out the acyltransferase‐encoding genes *mac1* and *mac2*, and the acetyltransferase‐encoding gene *mat1* on the putative BGC (Figure 3d). Among the mutants obtained, the Δ*mat1* strain selectively produced MEL‐D, while neither the Δ*mac1* nor the Δ*mac2* strains produced any MELs. Therefore, two acyltransferases are considered to be necessary for the production of MELs. (Hewald et al., 2006). In addition, this predicted BGC also contains a glycosyltransferase Emt1 and a membrane transporter Mmf1 (Figure 3d) (Hewald et al., 2006). A recent knockout experiment of *mmf1* demonstrated that Mmf1 is required as a transporter for the secretion of acetylated MELs without affecting the efflux of unacetylated MELs (Becker et al., 2022). Combined with the biosynthesis of MELs in other fungi, the biosynthesis of MELs in *U. maydis* is as follows: Emt1 catalyses the condensation of mannose and erythritol to form disaccharides, and Mac1 and Mac2 are responsible for the esterification of disaccharides. The esterified disaccharide is then acetylated by Mat1 to obtain MELs, which are transferred to the extracellular space by Mmf1 (Figure 3e) (Saika et al., 2018).

The biosynthetic gene clusters of MELs have been investigated in *U. maydis* in addition to being analysed, predicted, and identified in *Ustilago hordei* (Deinzer et al., 2019), *Pseudozyma antarctica* (Saika et al., 2018), and *Aspergillus nidulans* (Hewald et al., 2006). The BGCs of MELs from these various species show high homology (Figure 3d).

### 4.3 Siderophores and nonribosomal peptides

Most microorganisms produce a group of low‐molecular‐weight compounds called siderophores during iron stress, which have a particular affinity for iron acquisition and storage (Neilands, 1981). An evaluation of the role of siderophores on *U. maydis* in its pathogenicity showed that a high‐affinity iron uptake system involving siderophores is indispensable (Mei et al., 1993). Ferrochrome (8 in Figure 1) and ferrochrome A (9) are two siderophore derivatives produced by *U. maydis* (Haas et al., 2008; Yuan et al., 2001) that are cyclic hexapeptides consisting of three *δ*‐*N*‐acyl‐*N*‐hydroxy‐ornithine and three amino acids, glycine, serine, and alanine (Winkelmann, 2007; Yuan et al., 2001). The modification of the side chain of the ornithine residue and the difference in the species of the other three amino acids that form the cyclic peptide results in different siderophores (Bushley et al., 2008; Haas et al., 2008; Winkelmann, 2007).

**FIGURE 4:**
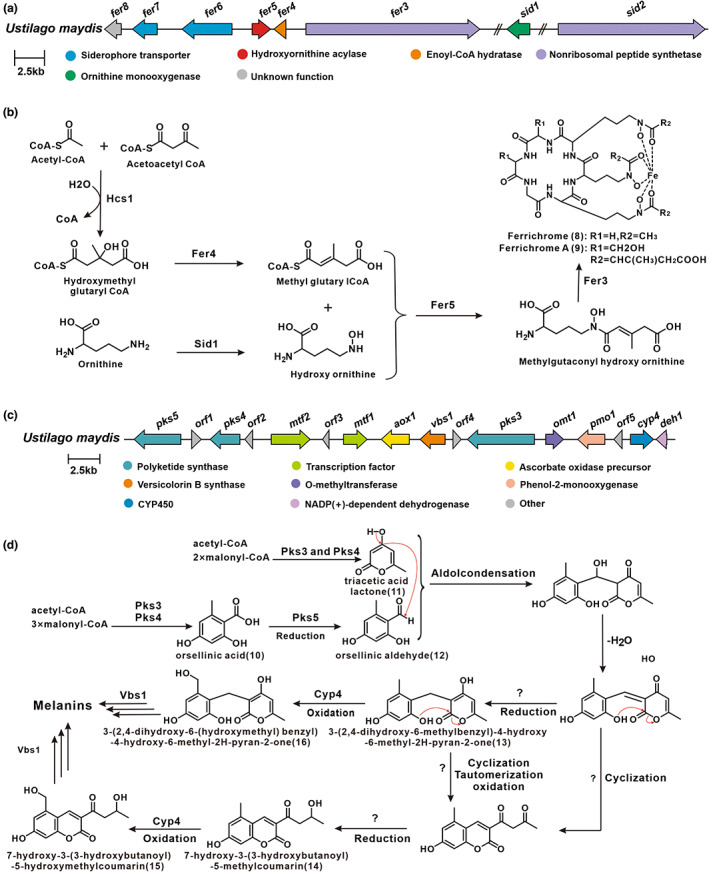
The (proposed) biosynthesis of ferrichromes and melanins. (a) Biosynthetic gene clusters (BGCs) for ferrichrome and ferrichrome A, (b) the proposed pathway of ferrichrome and ferrichrome A, (c) BGC for melanins, and (d) the proposed pathway of melanins.

Transcriptome analysis based on whole‐genome microarrays identified three gene clusters related to the high‐affinity iron uptake system and determined that the iron permease Fer2 plays a vital role in this system. Strains with normal *fer2* function have a selective growth advantage under iron (III)‐limiting conditions compared to strains with inactive *fer2*, thus suggesting that a high‐affinity iron uptake system based on permeases plays a key role in the virulence of *U. maydis* (Eichhorn et al., 2006). Ornithine monooxygenase Sid1 and non‐ribosomal peptide synthase Sid2 are the first identified functional genes for ferrichrome biosynthesis in *U. maydis*. Sid1 catalyses the first step in siderophore biosynthesis, and the inactivation of *sid1* results in a lack of ferrichrome production (Mei et al., 1993; Wang et al., 1989; Yuan et al., 2001). Subsequently, four new members involved in the biosynthesis of ferrichrome A in *U. maydis, fer3*, *fer4*, *fer5*, and *hcs1*, were discovered. These genes constitute the bulk of the BGC. This BGC encodes six functional proteins, a non‐ribosomal peptide synthase Fer3, an enoyl‐CoA hydratase Fer4, a hydroxyornithine acylase Fer5, two transporters Fer6 and Fer7, and a hypothetical gene, *fer8* (Figure 4a) (Winterberg et al., 2010). High‐performance liquid chromatography‐mass spectrometry (HPLC‐MS) analysis of the metabolites of individual mutants of *fer3*, *fer4*, and *fer5* found that their inactivation all resulted in the absence of ferrichrome A, which suggested that these three genes are required for ferrichrome A production. Overexpression of the HMG‐CoA synthase Hcs1 in *U. maydis* leads to an increase in ferrichrome A production. In‐depth analysis revealed that Hsc1 associates with Fer4, implying that the product of Hsc1 is a substrate for Fer4 (Winterberg et al., 2010).

A clear pathway for ferrichrome biosynthesis is deduced in the diagram. Hcs1 catalyses the formation of HMG‐CoA from acetyl‐CoA and acetoacetyl‐CoA. HMG‐CoA is further catalysed by Fer4 to methylglutamyl‐CoA. Simultaneously, the hydroxyornithine and methylglutamyl‐CoA generated from ornithine catalysed by Sid1 are used as substrates to form methylgutaconyl hydroxy ornithine catalysed by Fer5. Finally, methylgutaconyi hydroxy ornithine is cyclized with specific amino acids by Fer3 to form ferrichromes (Figure 4b) (Winterberg et al., 2010).

### 4.4 Melanin and polyketides

The formation of spores in plant tumour‐like tissues was found to be associated with melanin (Islamovic et al., 2015). The production of melanin in *U. maydis* is catalysed by the laccase Lac1 and the polyketide synthase PKS1 during tumour formation (Islamovic et al., 2015). Additional PKSs also contribute to melanin formation, which is present on chromosome 12 and expressed during late stages of infection (Lanver et al., 2018).

Reyes‐Fernandez et al. (2021) identified novel melanin associated with polyketide synthases in *U. maydis*, and these PKSs synthesize orsellinic acid (OA, 10 in Figure 1), triacetic acid lactone (TAL, 11), and OA derivatives (12–16), as well as further forming coumarin and pyran‐2‐one. Coumarin and pyran‐2‐one are the precursors of this new melanin, whereas the precursors of most fungal melanins are 1,8‐dihydroxynaphthalene (DHN) or L‐3,4‐dihydroxyphenylalanine (L‐DOPA). This finding demonstrates the complexity of melanin synthesis in *U. maydis*. It serves as a reference for the structural identification of melanin (Reyes‐Fernandez et al., 2021).

Melanins polymerized as DHN or L‐DOPA units are common melanins in fungi, with the former being more prevalent (Tsai et al., 1999; Woo et al., 2010). The discovery of two PKSs in *U. maydis* has been hypothesized to be associated with the melanization of teliospores (Islamovic et al., 2015). Reyes‐Fernández et al. (2021) discovered an unusual melanin biosynthetic pathway in *U. maydis* that uses polyketide synthases to produce melanin precursors. The BGC codes for seven functional proteins include three polyketide synthases (Pks3, Pks4, and Pks5), a P450 oxidase (Cyp4), a versicolorin B synthase (Vbs1), and two transcription factors (Mtf1 and Mtf2) (Figure 4c). Overexpression experiments demonstrated that the transcription factor Mtf1 regulates melanin synthesis and indicated that this gene cluster is a silent BGC. Three genes encoding PKS, *pks3, pks4*, and *pks5*, are present in the telomeric region of chromosome 12. They were found to be part of the biosynthetic gene cluster by gene knockout. Pks3 and Pks4 work together as heterodimers to generate OA and TAL, which is then reduced to orsellinic aldehyde by Pks5. Then, TAL and orsellinic aldehyde undergo spontaneous aldol condensation to form a specific intermediate, which undergoes reduction, oxidation, and tautomerization to form the melanin precursor, and finally generates melanin under the catalysis of Vbs1 (Figure 4d) (Reyes‐Fernandez et al., 2021). The formation of such complex multi‐PKS‐involved polymers expands the complexity of PKS functions and offers new insights into PKS evolution.

### 4.5 Miscellaneous compounds

Reineke et al. (2008) proved that *U. maydis* could efficiently produce indole‐3‐acetic acid (IAA, 17 in Figure 1) from tryptophan (Trp), thus confirming the prediction that *U. maydis* could synthesize IAA (Moulton, 1942). Although the synthesis of IAA by *U. maydis* is enhanced when maize plants are infected, the evidence that the IAA produced by *U. maydis* was involved in tumour formation was still minimal: *U. maydis* could still induce tumour formation despite the simultaneous knockout of four essential genes, *tam1*, *tam2*, *iad1*, and *iad2*, required for IAA biosynthesis (Doehlemann, Wahl, Vranes, et al., 2008; Reineke et al., 2008). In addition, *U. maydis* was found to efficiently convert exogenously added Trp to IAA (Basse et al., 1996).

Plant hormones produced or modified by microorganisms have been shown to play a role in plant disease occurrence. *U. maydis* produces two phytohormones, abscisic acid (ABA, 18) and cytokinins (CKs) (Morrison et al., 2015), with the former playing a role in the growth of *U. maydis* (Mills & Staden, 1978; Moulton, 1942). No fewer than 10 CKs have been identified from *U. maydis* (Morrison et al., 2015); a representative CK (19) is shown in Figure 1. A survey of CK and ABA levels during *U. maydis* pathogenesis clearly shows that CKs and ABA can accumulate in *U. maydis*‐infected maize tissues and that levels are higher in tumours formed by *U. maydis* than in other maize tissues (Bruce et al., 2010).

Indole pigments such as pityriacitrin (20), pityrianhydride (21), and pityriarubins A–C (22–24) are small molecular compounds found in the tumour tissue of *U. maydis*. Their content increases with the size of the tumour tissue (Jiménez‐Bremont et al., 2014; Rodriguez‐Kessler et al., 2008). *U. maydis* also produces itaconate (25), an essential unsaturated organic acid with high commercial value (Okabe et al., 2009; Willke & Vorlop, 2001). Whether or not itaconate (25) is involved in infection remains unknown, despite the literature claiming that organic acids contribute to smut infection (Kretschmer et al., 2022).

## 5 DISCUSSION AND PERSPECTIVES

The dimorphic life cycle of crop smut is naturally related to the process of infecting maize, starting from the adhesion of appressoria to the maize surface to the formation of new spores in tumour tissue. This part of the cycle of parasitizing maize plants demonstrates the mycelial shape. The interaction process between the mycelium‐like smut and maize has been profoundly studied at the cellular and molecular levels. The effectors secreted by *U. maydis* inhibit the rearrangement activity of maize due to infection and facilitates its infection. *U. maydis* additionally assists the adhesion of appressoria to the surface of plants by producing lipopolysaccharide compounds with surfactant properties. Genome sequencing of multiple *U. maydis* isolates will further facilitate the discovery of more effectors. It will also provide favourable conditions for the biosynthesis of pathogenicity‐related secondary metabolites.

Structurally diverse secondary metabolites of *U. maydis*, including UAs, MELs, ferrichromes, and melanin, have been found to be extensively involved in the pathogenicity process of *U. maydis*. These compounds have also been shown to have antibacterial and antitumour properties, antioxidant activities, and biosurfactant and other biological functions. It is often considered that secondary metabolites are not of much significance to their producers. However, this is not absolute, as in the case of *U. maydis* and its secondary metabolites.

It has been shown that as far as the structural type of secondary metabolites is concerned, *U. maydis* as a basidiomycete differs significantly from that of mushroom‐form basidiomycete species like *Laetiporus* spp. (Duan et al., 2022) and *Inonotus hispidus* (Wang et al., 2022), and the number of secondary metabolites is much less. Although the genome of *U. maydis* is predicted to contain multiple BGCs for secondary metabolites (Figure S1), not many compounds have been isolated from *U. maydis*. This implies that most BGCs of *U. maydis* are inactive, which is also supported by the silent BGCs of some metabolites in *U. maydis* found in the few biosynthetic studies.

In conclusion, this work summarizes the progress of pathogenicity studies, effectors and their assistance in pathogenesis, as well as secondary metabolites involved in the pathogenic process and their biosynthesis by *U. maydis*. It summarizes the pathogenesis and players of crop smut from a unique perspective, and provides systematic insights for understanding the pathogenicity.

## CONFLICT OF INTEREST STATEMENT

The authors declare that the research was conducted without any commercial or financial relationships that could be construed as a potential conflict of interest.

## Supporting information


**Figure S1** The identified biosynthetic gene clusters (BGCs) and predicted BGCs in *Ustilago maydis*.

**Table S1** Other effectors associated with the pathogenesis of *Ustilago maydis*.

**Table S2** Comparison of genome quality of *Ustilago maydis*.

## Data Availability

Data sharing is not applicable to this article as no new data were created or analysed.
