# Supplementary material for: Progress in pathogenesis research of Ustilago maydis, and the metabolites involved along with their biosynthesis
Source: Mol Plant Pathol. 2023 Feb 17;24(5):495–509. doi: 10.1111/mpp.13307 (PMC10098057; doi:10.1111/mpp.13307)
Supplement: Supplementary file 1 — Figure S1 The identified biosynthetic gene clusters (BGCs) and predicted BGCs in Ustilago maydis. [file MPP-24-495-s003.docx]

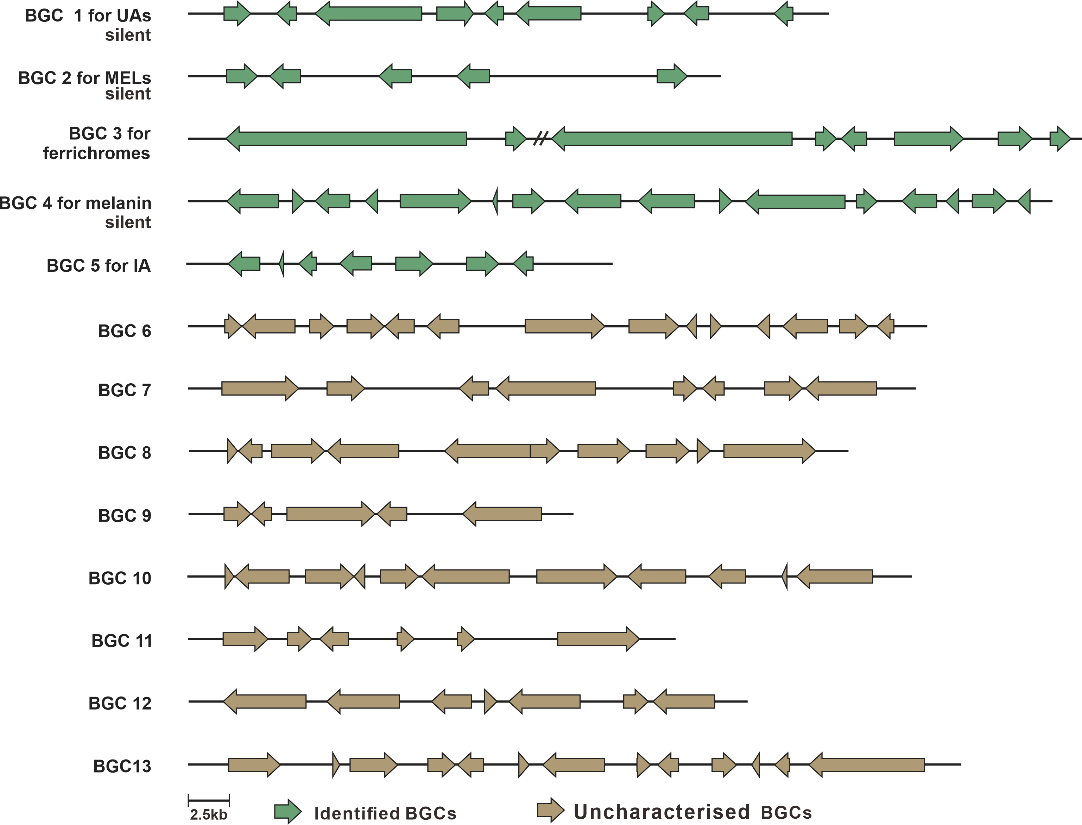


**Fig. S1 The identified BGCs and predicted BGCs in *U. maydis*.**

The gene cluster prediction is performed with antiSMASH (https://fungismash.secondarymetabolites.org).
