# Supplementary material for: Progress in pathogenesis research of Ustilago maydis, and the metabolites involved along with their biosynthesis
Source: Mol Plant Pathol. 2023 Feb 17;24(5):495–509. doi: 10.1111/mpp.13307 (PMC10098057; doi:10.1111/mpp.13307)
Supplement: Supplementary file 2 — Table S1 Other effectors associated with the pathogenesis of Ustilago maydis. [file MPP-24-495-s002.docx]

**Table S1. Other effectors associated with the pathogenesis of *U. maydis*.**

| Entry | Effector | Function/Feature/Note | Reference |
| --- | --- | --- | --- |
| ApB73 | Apathogenic in B73 | It was identified by transcriptome analysis and secreted into the interface of biological nutrition. | [1] |
| Hum3 | - | Identified according to its specific sequence or structure. | [2] |
| Rsp1 | repetitive and secreted protein 1 |  | [3] |
| Sho1 | synthetic high osmolarity sensitive 1 | Sensing signals such as hydrophobic structures and cutin monomers on plant surfaces. | [4, 5] |
| Msb2 | multicopy suppressor of a budding defect 2 |  |  |
| Pmt | protein mannose transferase | Promote the ability of hyphae to form attachment cells and penetrate the stratum corneum of plants. | [6] |
| Spp1 | signal peptide peptidase 1 | Spp1 is the only signal peptide peptidase of *U. maydis.* | [7] |
| Clp1 | clampless 1 | Clp1 is an important protein regulating cell cycle, which can promote the replication and proliferation of *U. maydis*. | [8] |
| Cib1 | Clp1 interacting bZip1 | Clp1/Cib1 complex can be regarded as a checkpoint for *U. maydis* to start parasitic life. | [8] |
| Fly1 | fungalysin1 | Chitinase that destroys host plants | [9] |
| Sta1 | small tumor-associated 1 | It was synthesized only when *U. maydis* budded, and the secreted Sta1 protein could not bind to the surface of haploid, but could be adsorbed on the cell wall of binucleate hyphae. | [10] |
| Scp2 | sterol carrier protein 2 | Scp2 is located in the haploid peroxisome, and its function is unclear. | [11] |
| Erc1 | enzyme required for cell-to-cell extension | Enzymes required for cell-to-cell extension contribute to the virulence of *U. maydis* on maize leaves, but not on ears. | [12] |
| Stp1/2/3 | _ | Secreted by *U. maydis* hyphae in glycosylated form, mainly confined to hyphae that proliferate in epidermis. | [13] |
| UmFly1 | *Ustilago maydis* fungalysin | Corn chitinase ZmChiA can be cracked to reduce its cracking activity and protect its cell wall from plant chitinase. | [9] |
| Tay1 | Taygeta1 | Mer1 and Tay1 are soluble proteins secreted  by *U. maydis* into the biotrophic interphase upon host colonization. The former is located in the nucleus and the latter in the cytoplasm. | [14] |
| Mer1 | Merope1 |  | [14] |

“-” indicates not available.

**Reference**

1. Stirnberg, A. and A. Djamei, *Characterization of ApB73, a virulence factor important for colonization of Zea mays by the smut Ustilago maydis.* Molecular Plant Pathology, 2016. **17**(9): p. 1467-1479.

2. Mueller, O., et al., *The secretome of the maize pathogen Ustilago maydis.* Fungal Genetics and Biology, 2008. **45 Suppl 1**: p. S63-70.

3. Matei, A., et al., *How to make a tumour: cell type specific dissection of Ustilago maydis-induced tumour development in maize leaves.* New Phytologist, 2018. **217**(4): p. 1681-1695.

4. Lanver, D., et al., *Plant surface cues prime Ustilago maydis for biotrophic development.* PLoS pathogens, 2014. **10**(7): p. e1004272.

5. Lanver, D., et al., *Sho1 and Msb2-related proteins regulate appressorium development in the smut fungus Ustilago maydis.* The Plant Cell, 2010. **22**(6): p. 2085-2101.

6. Fernández-Álvarez, A., et al., *Identification of O-mannosylated Virulence Factors in Ustilago maydis.* PLoS Pathogens, 2012. **8**: p. e1002563.

7. Voss, M., et al., *Shedding of glycan-modifying enzymes by signal peptide peptidase-like 3 (SPPL3) regulates cellular N-glycosylation.* The EMBO Journal, 2014. **33**(24): p. 2890-2905.

8. Heimel, K., et al., *The Ustilago maydis Clp1 Protein Orchestrates Pheromone and b-Dependent Signaling Pathways to Coordinate the Cell Cycle and Pathogenic Development.* The Plant Cell, 2010. **22**(8): p. 2908-2922.

9. Ökmen, B., et al., *Dual function of a secreted fungalysin metalloprotease in Ustilago maydis.* New Phytologist, 2018. **220**(1): p. 249-261.

10. Tanaka, S., et al., *The functionally conserved effector Sta1 is a fungal cell wall protein required for virulence in Ustilago maydis.* New Phytologist, 2020. **227**(1): p. 185-199.

11. Krombach, S., et al., *Virulence function of the Ustilago maydis sterol carrier protein 2.* New Phytologist, 2018. **220**(2): p. 553-566.

12. Ökmen, B., et al., *A conserved enzyme of smut fungi facilitates cell-to-cell extension in the plant bundle sheath.* Nature Communications 2022. **13**(1): p. 6003.

13. Schipper, K., *Charakterisierung eines Ustilago maydis Genclusters, das für drei neuartige sekretierte Effektoren kodiert.* 2009.

14. Navarrete, F., et al., *The Pleiades are a cluster of fungal effectors that inhibit host defenses.* PLoS Pathog, 2021. **17**(6): p. e1009641.
