# Supplementary material for: Progress in pathogenesis research of Ustilago maydis, and the metabolites involved along with their biosynthesis
Source: Mol Plant Pathol. 2023 Feb 17;24(5):495–509. doi: 10.1111/mpp.13307 (PMC10098057; doi:10.1111/mpp.13307)
Supplement: Supplementary file 3 — Table S2 Comparison of genome quality of Ustilago maydis. [file MPP-24-495-s001.docx]

**Table S2. Comparison of genome quality of *U. maydis*.**

| **Strains** | **Genome coverage** | **No. of**  **contigs** | **Scaffold/Contig**  **N50** | **Genome size**  **(Base pairs)** | **GC content**  **(%)** | **GenBank assembly accession** |
| --- | --- | --- | --- | --- | --- | --- |
| *U. maydis* 521 | 10.0x | 254 | 884,984 | 19,664,356 | 53.96 | GCA_000328475.2 |
| *U. maydis* FB1 | 80.0x | 365 | 884,922 | 19,662,687 | 53.97 | GCA_016617945.1 |
| *U. maydis* FB2 | 80.0x | 398 | 884,902 | 19,662,461 | 53.95 | GCA_016617935.1 |
| *U. maydis* 482-v1 | 40x | 46 | 876,010 | 20,585,367 | 53.90 | GCA_928722285.1 |
| *U. maydis* 485 | 40x | 43 | 743,668 | 20,582,581 | 53.77 | GCA_928722245.1 |
| *U. maydis* 482-v2 | 40x | 68 | 674,905 | 20,591,394 | 53.85 | GCA_928722265.1 |
| *U. maydis* 198 | 40x | 107 | 660,941 | 21,122,121 | 53.41 | GCA_928743665.1 |
| *U. maydis* JCM2005 | 173x | 104 | 805,038 | 19,606,871 | 53.88 | GCA_001599495.1 |
| *U. maydis* I5 | 165x | 395 | 521,084 | 19,572,093 | 54.06 | GCA_018154835.1 |
| *U. maydis* O4 | 142x | 356 | 551,770 | 19,554,160 | 53.99 | GCA_018154565.1 |
| *U. maydis* P2 | 129x | 349 | 469,913 | 19,523,824 | 53.92 | GCA_018154495.1 |
| *U. maydis* I3 | 171x | 414 | 551,920 | 19,536,456 | 53.87 | GCA_018154895.1 |
| *U. maydis* T2 | 114x | 528 | 375,987 | 19,647,002 | 54.04 | GCA_018154095.1 |
| *U. maydis* P5 | 138x | 455 | 475,227 | 19,546,661 | 53.94 | GCA_018154295.1 |
| *U. maydis* S3 | 120x | 481 | 498,675 | 19,619,605 | 53.97 | GCA_018154185.1 |
| *U. maydis* S2 | 185x | 486 | 509,925 | 19,543,733 | 53.93 | GCA_018154205.1 |
| *U. maydis* FBA | 100.0x | 175 | 292,465 | 19,488,417 | 54.05 | GCA_023212765.1 |
| *U. maydis* P4 | 109x | 354 | 444,704 | 19,510,621 | 53.91 | GCA_018154395.1 |
| *U. maydis* P3 | 110x | 518 | 498,918 | 19,911,006 | 52.92 | GCA_018154425.1 |
| *U. maydis* T6 | 108x | 439 | 498,693 | 19,553,394 | 53.96 | GCA_018153955.1 |
| *U. maydis* T5 | 125x | 538 | 376,757 | 19,603,521 | 54.08 | GCA_018153985.1 |
| *U. maydis* S5 | 149x | 442 | 499,238 | 19,506,874 | 53.96 | GCA_018154105.1 |
| *U. maydis* O1 | 115x | 401 | 406,270 | 19,500,729 | 54.02 | GCA_018154715.1 |
| *U. maydis* O3 | 126x | 405 | 446,121 | 19,573,542 | 54.05 | GCA_018154645.1 |
| *U. maydis* P6 | 132x | 427 | 468,461 | 19,536,400 | 53.88 | GCA_018154385.1 |
| *U. maydis* O5 | 113x | 413 | 470,405 | 19,511,902 | 54.04 | GCA_018154595.1 |
| *U. maydis* I2 | 116x | 1,016 | 378,139 | 19,792,732 | 53.88 | GCA_018154905.1 |
| *U. maydis* O2 | 113x | 457 | 356,334 | 19,516,151 | 54.00 | GCA_018154685.1 |
| *U. maydis* T4 | 120x | 583 | 330,199 | 19,547,534 | 54.01 | GCA_018154035.1 |
| *U. maydis* I6 | 125x | 500 | 345,526 | 19,536,098 | 53.97 | GCA_018154785.1 |
| *U. maydis* I4 | 192x | 1,664 | 318,866 | 21,059,893 | 51.05 | GCA_018154945.1 |
| *U. maydis* ATCC 22904 | 80.0x | 2,695 | 120,656 | 20,132,962 | 53.98 | GCA_001736215.1 |
| *U. maydis* AB33P5deltaR | 80.0x | 1,978 | 111,545 | 19,929,430 | 54.03 | GCA_001736185.1 |
| *U. maydis* ATCC bA22899 | 80.0x | 2,938 | 109,028 | 20,208,930 | 53.99 | GCA_001662005.1 |
| *U. maydis* RK818 | 80.0x | 4,767 | 104,350 | 20,622,051 | 53.97 | GCA_001660065.1 |
| *U. maydis* ATCC 22901 | 80.0x | 2,505 | 102,739 | 20,063,391 | 53.97 | GCA_001736155.1 |
| *U. maydis* FB2CGL-UMa1502 | 34x | 646 | 83,229 | 19,437,552 | 54.19 | GCA_013387245.1 |
| *U. maydis* FB2CGL-UMa1795 | 31x | 706 | 74,590 | 19,430,330 | 53.99 | GCA_013387255.1 |
